# Butorphanol-midazolam combination therapy for the treatment of intracranial hypertension in a patient with tuberculous meningitis: a case study

**DOI:** 10.1186/2193-1801-2-442

**Published:** 2013-09-08

**Authors:** Hiroshi Kataoka, Satoshi Ueno

**Affiliations:** Department of Neurology, Nara Medical University, 840 Shijo-cho, Kashihara, Nara, 634-8522 Japan

**Keywords:** Tuberculous meningitis, Intracranial hypertension, Headache, Midazolam, Butorphanol

## Abstract

**Introduction:**

Intracranial hypertension, which often occurs in patients with tuberculous meningitis, is associated with high morbidity and mortality. We describe a patient with tuberculous meningitis who had intracranial hypertension -induced fulminant headache that responded to intravenous butorphanol-midazolam combination therapy.

**Case presentation:**

A 50-year-old woman with a fever and headache for 24 days was given a diagnosis of tuberculous meningitis on the basis of the results of polymerase chain reaction amplification and Ziehl-Neelsen staining. Headache with vomiting developed despite administration of steroids, osmotic, and antituberculosis treatments. The patient was admitted in a confusional state. The initial pressure (420 mmHg) in cerebrospinal fluid was increased. She was given intravenous mannitol, dexamethasone, pentazocine and diazepam, or she was sedated with propofol, with no response. Next, a combination of butorphanol and midazolam was infused intravenously and finally resolved the confusional state. The initial pressure decreased, and she no longer complained of headache requiring medication.

**Discussion and evaluation:**

The butorphanol-midazolam combination therapy may have reduced intracranial pressure, leading to down-regulation of headache. Sedation induced by such combination of drugs was not accompanied by amnesia or impaired psychomotor function.

**Conclusions:**

The butorphanol-midazolam combination therapy might be an option for the management of intracranial hypertension in central nervous system infections.

## Introduction

Tuberculosis is one of the most devastating infectious diseases worldwide. The global incidence of tuberculosis peaked around 2004 and has decreased at a rate of less than 1% per year (Lawn and Zumla 2011). However, the incidence and prevalence of tuberculosis is high in most developing countries, including those in Asia (Lawn and Zumla 2011). At present, the incidence of tuberculosis in Japan is lower than that in developing countries in Asia (Lawn and Zumla 2011), but is higher among older people as compared with Europe and North American (Global Tuberculosis Control 2008 2013). Central nervous system involvement, while rare, is the most severe form of tuberculosis. Reportedly, about 10 percent of patients with tuberculosis have central nervous system involvement (Murthy 2005). Tuberculous meningitis (TBM) is primarily a disease of the meninges, which can cause edema, infarction, or hydrocephalus, and multiple pathological changes lead to elevated intracranial pressure (Murthy 2005). Intracranial hypertension (ICH), which often occurs in patients with TBM, is associated with high morbidity and mortality (Murthy 2005). ICH can be managed by osmotic treatment with mannitol or hypertonic saline or by anti-inflammatory and immunomodulatory treatment with steroids. ICH must be treated with these options in addition to antituberculosis therapy to avoid complications; however, surgical intervention including ventriculo-peritoneal shunt is a second option reserved for patients who do not respond to pharmacological therapy (Murthy 2005). We describe a patient with TBM who had ICH-induced fulminant headache that responded to intravenous butorphanol-midazolam combination therapy. This case suggests that this combination therapy can be an alternative treatment for the management of ICH in patients who do not respond to osmotic therapy or steroids, which have been recommended for the medical management of ICH associated with tuberculous meningitis.

## Case presentation

**Figure 1:**
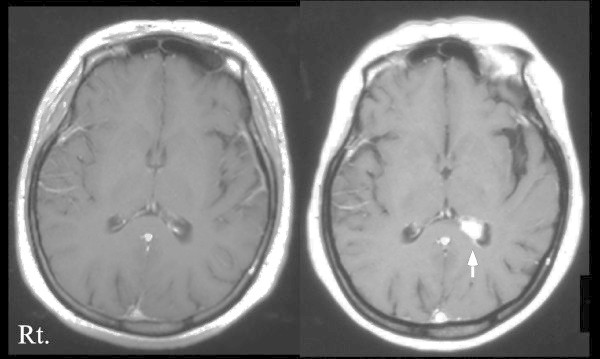
**Contrast T1-weighted magnetic resonance images (MRI).** Initial MRI showed no abnormal enhancement (left panel), but follow-up MRI revealed enhancement of the choroid plexus and posterior horn of the lateral ventricle, with no ventricular enlargement (right panel).

A 50-year-old previously healthy Japanese woman who had a fever and headache for 24 days was admitted to our hospital. Initial examination confirmed a fever (39.4°C) and frontal tension-type headache. She had no history of travel overseas for several years or of pulmonary tuberculosis. Her consciousness was clear, with no neurologic deficits. Meningismus and papilledema were absent. Blood cell counts and the results of routine biochemical analysis were normal, expect for glucose (117 mg/dl). Human immunodeficiency virus was negative. Cranial computed tomography (CT) revealed a spotty calcification in the pons. Lumbar puncture on day 1 showed an initial pressure of 220 mmHg, 173 white cells/mm^3^ (98% lymphocytes), a protein concentration of 160 mg/dl, and a glucose concentration of 40 mg/dl. Intravenous acyclovir (10 mg/kg/day, 17 days) and glycerin (40 mg/day) were started for a suspected diagnosis of viral encephalitis. Cranial enhanced magnetic resonance imaging (MRI) on day 6 revealed no abnormal high-signal intensity or enhancement (Figure 1). On day 18, intravenous acyclovir was prescribed because a high fever with headache persisted, and the initial pressure and white cell count in CSF had increased to 335 mmHg and 380 white cells/mm^3^, respectively. The results of polymerase chain reaction amplification and Ziehl-Neelsen staining were positive for *Mycobacterium tuberculosis* in cerebrospinal fluid (CSF) on day 18. Isoniazid (300 mg/day), rifampicin (450 mg/day), pyrazinamide (1.5 g/day), and ethambutol (750 mg/day) were started concurrently with intravenous prednisolone (30 mg/day), the dose of which was tapered. During these antituberculosis treatments, the tension-type headache responded to diclofenac sodium or intravenous glycerin. However, the headache changed to posterior cervical pain accompanied by a high fever and meningismus on day 28. Repeated cranial enhanced MRI showed enhancement of the choroid plexus and posterior horn of the lateral ventricle, with no ventricular enlargement (Figure 1). On day 55, frontal headache developed in addition to the posterior cervical pain, often interrupting sleep and not responding to diclofenac sodium or intravenous glycerin. Meningismus persisted, and abducens paralysis was present. Repeated CSF examination showed that white cell count (200/mm^3^) had decreased in response to antituberculosis drugs, but the initial pressure (420 mmHg) and protein concentration (338 mg/dl) had increased, and the glucose concentration (30 mg/dl) had deceased. There was no evidence of hydrocephalus on a CT scan. Mannitol (120 mg/day) and dexamethasone (8 mg/day) with taper were started in addition to antituberculosis drugs and glycerin (20 mg/day). On day 66, headache with vomiting developed despite continued treatment. She writhed, turned her body, and moved her limbs violently on the bed, crying out “painful, painful.” The patient was admitted in a confusional state, which could be coped with by her family. The score on the Glasgow Coma Scale (GCS) was 13. Cranial CT showed the same findings as previously, without hydrocephalus. On day 67, headache with vomiting and a confusional state, associated with removing clothes or extracting the intravenous line, further intensified, which constantly required surveillance by a physician or nurse. She was given several intravenous injections of pentazocine (7.5 mg) and diazepam (2.5 mg), with no response. The patient was sedated with propofol under mechanical ventilation because her respiratory function was suppressed by the previous injections. Intravenous propofol produced sedation for several hours, but even after increasing to the maximum dose of propofol (2.8 mg/kg/hr), the confusional state and high fever (≧ 38°C) did not resolve. Papilledema was evident. On day 68, we switched from intravenous propofol to intravenous (2.0 ml/hr) combination therapy (total 40 ml) with butorphanol (20 mg) and midazolam (100 mg) in saline solution (10 ml). This butorphanol-midazolam combination therapy was given for 16 days (maximum dose, 3.0 ml/hr), and on day 1 of combination therapy the confusional state resolved. Subsequently, the patient was in a deeply sedated state while receiving mechanical ventilation, and the GCS score was 3. On day 69, the initial pressure (240 mmHg) and white cell count (195/mm^3^) had decreased. On day 73, she could nod in response to an easy question, and the body temperature rose only to 37.5°C. On day 78, intraspinal injections of isoniazid (100 mg per time) were started three times per week with taper and were continued for 5 months. On day 84, the butorphanol- midazolam combination therapy and mechanical ventilation were withdrawn, her consciousness was clear. The GCS score was 15, and she no longer complained of headache requiring medication. CSF analysis on day 87 showed further decreases in initial pressure (110 mmHg), white cell count (48/mm^3^), and protein level (117 mg/dl) and an increased glucose concentration (43 mg/dl). Seven months after admission, she was discharged from our hospital and subsequently received antituberculosis drugs for 14 months, resulting in complete recovery.

## Discussion

This report describes a patient with intolerable, refractory, fulminant headache caused by ICH without hydrocephalus. The headache finally responded to intravenous butorphanol-midazolam combination therapy. To our knowledge, this is the first time to document the use of this combination therapy in a patient with severe headache.

ICH is well known to frequently cause headache (Murthy 2005). TBM is primarily a disease of the meninges associated with multiple potential causes of ICH, such as cerebral edema, hydrocephalus, infarction, adhesion formation of the basal subarachnoid cisterns, or tuberculoma (Murthy 2005). These changes were not found on CT or MRI studies in our patient, except for enhancement of the colloid plexus. We speculate that an imbalance between CSF production and absorption caused by the colloid plexus lesions may have contributed to ICH, leading to the fulminant headache.

Osmotic therapy or steroids have been recommended for the medical management of ICH associated with TBM (Murthy 2005), but these treatments failed to resolve the intolerable, refractory, fulminant headache caused by ICH without hydrocephalus in our patient. We tried high-dose intravenous propofol, but adequate sedation could not be induced because of the intense headache. Intravenous butorphanol-midazolam combination therapy was finally able to control the headache. Midazolam or butorphanol is widely used as a sedative or analgesic, and the combination of these two medications is also useful for sedation (Dershwitz et al. 1991). Midazolam is a benzodiazepine derivative that stimulates γ-aminobutyric acid-A (GABA-A) receptors by enhancing channel opening (Bai et al. 1999), and its agonist activity leads to generalized inhibition of neuronal firing (Enna and Mohler 1987). Similar to midazolam, propofol is effective for the management of refractory headache (Mendes et al. 2002). Butorphanol is believed to act as a partial agonist at kappa opioid receptors (Wood et al. 1983), and the resulting kappa-type opioid produces sedation (Abboud et al. 1987). The mechanism by which butorphanol-midazolam combination therapy alleviated headache remains uncertain, but may have involved a reduction in intracranial pressure or down-regulation of headache. The consciousness of our patient was clear after the withdrawal of the combination therapy. Sedation induced by such combination of drugs was not accompanied by amnesia or impaired psychomotor function (Dershwitz et al. 1991), and the combination therapy may have no neurophysiological effect when used as a supplement to propofol-induced anesthesia.

## Conclusions

Butorphanol-midazolam combination therapy may be useful for ICH-induced fulminant headache in patients who do not respond to recommended osmotic or steroids treatments for ICH. The efficacy of low-dose butorphanol-midazolam combination therapy without the need for mechanical ventilation is unknown, but the treatment might be an option for the management of ICH or ICH-induced headache because of potent analgesic and anxiolytic efficacy in patients with central nervous system infections.

## Consent

Written informed consent was obtained from the patient for publication of this case and any accompanying images.
